# Intervertebral Disc-Derived Stem/Progenitor Cells as a Promising Cell Source for Intervertebral Disc Regeneration

**DOI:** 10.1155/2018/7412304

**Published:** 2018-12-18

**Authors:** Binwu Hu, Ruijun He, Kaige Ma, Zhe Wang, Min Cui, Hongzhi Hu, Saroj Rai, Baichuan Wang, Zengwu Shao

**Affiliations:** ^1^Department of Orthopaedics, Union Hospital, Tongji Medical College, Huazhong University of Science and Technology, Wuhan 430022, China; ^2^National Trauma Center, National Academy of Medical Sciences, Kathmandu, Nepal

## Abstract

Intervertebral disc (IVD) degeneration is considered to be the primary reason for low back pain. Despite remarkable improvements in both pharmacological and surgical management of IVD degeneration (IVDD), therapeutic effects are still unsatisfactory. It is because of the fact that these therapies are mainly focused on alleviating the symptoms rather than treating the underlying cause or restoring the structure and biomechanical function of the IVD. Accumulating evidence has revealed that the endogenous stem/progenitor cells exist in the IVD, and these cells might be a promising cell source in the regeneration of degenerated IVD. However, the biological characteristics and potential application of IVD-derived stem/progenitor cells (IVDSCs) have yet to be investigated in detail. In this review, the authors aim to perform a review to systematically discuss (1) the isolation, surface markers, classification, and biological characteristics of IVDSCs; (2) the aging- and degeneration-related changes of IVDSCs and the influences of IVD microenvironment on IVDSCs; and (3) the potential for IVDSCs to promote regeneration of degenerated IVD. The authors believe that this review exclusively address the current understanding of IVDSCs and provide a novel approach for the IVD regeneration.

## 1. Introduction

Low back pain (LBP) is one of the most common musculoskeletal disorders causing a tremendous socioeconomic burden to the patients due to lost productivity and increasing health care costs [1–3]. Although numerous and complex causes are involved in the pathogenesis of LBP, the intervertebral disc (IVD) degeneration appears to be the foremost cause [4, 5]. However, established treatments of IVD degeneration (IVDD), including medical and surgical treatments, are mainly focused on alleviating the symptoms rather than treating the underlying cause or restoring the structure and biomechanical function of the IVD [6–8].

The loss of disc cell viability and functionality plays a critical role in disturbing disc homeostasis, which reduces biosynthesis of extracellular matrix (ECM) components and triggers the IVDD [9, 10]. Therefore, cell-based therapy and regenerative medicine aiming at restraining or even reverting the loss of disc cell number and function have attracted much attention in the field of IVD regeneration [11]. Currently, a number of therapeutic modalities, such as growth factor supply, gene therapy and the delivery of functional cells, have been developed in order to rescue the disc cells [12–15]. Of these, the delivery of functional cells is, possibly, a promising therapeutic strategy. Many different kinds of functional cells from different areas of the body, i.e., nucleus pulposus cells (NPCs), bone marrow mesenchymal stem cells (BMSCs), adipose stem cells (ASCs), muscle-derived stem cells, synovial stem cells, induced pluripotent stem cells, olfactory neural stem cells, hematopoietic stem cells, and embryonic stem cells, can be successfully transplanted into the IVD with a hope to repair or regenerate the IVD [16]. Owing to wide availability and multilineage differentiation potential, the stem cells (SCs) have been extensively used and have shown a promising result in animal models and clinical trials [17, 18]. However, some obstacles are always hindering the further application of SCs in disc regeneration. These problems include puncture injury during SC extraction from the tissues and formation of osteophytes in the degenerated disc due to the leakage of SCs [19, 20]. Moreover, the microenvironment of IVD is characterized by excessive mechanical loading, high osmolarity, limited nutrition, acidic pH, and low oxygen tension [21–23]. Such microenvironment might impair the viability, proliferation, and ECM biosynthesis abilities of transplanted SCs leading to a limited repair potential [21–23]. Thus, it is desperately necessary to identify novel cell sources for IVD regeneration.

Many tissues have been identified to contain adult tissue-specific SCs, also known as endogenous SCs [24–26]. These endogenous SCs are capable of balancing the homeostasis of the tissues by regulating their own proliferation and differentiation. Therefore, endogenous stem/progenitor cells are regarded as a promising cell source for regenerating tissues because of the potential of overcoming the obstacles related to cell transplantation [24]. The IVD is the largest avascular structure in the body, which has been previously thought to have a little or poor self-repair capacity in adult mammals [27]. Nevertheless, many previous studies have indicated that the resident SCs exist both in normal and degenerated IVD and are referred to as IVD-derived stem/progenitor cells (IVDSCs) [28–31]. These cells can be isolated from different compartments of IVD, including nucleus pulposus (NP), annulus fibrosus (AF), and cartilage endplate (CEP) and can express most of the phenotype markers that define MSCs [29, 32–36]. Furthermore, it is also proven that there exists SC niche (SCN) within the IVD, which is confined around the perichondrium region adjacent to the epiphyseal plate (EP) and outer zone of the AF [6, 27]. Thus, promoting self-repair via mobilizing the endogenous SCs might be a prospective approach for stem cell-based therapy and the IVD regeneration. However, as a novel cell subset in IVD, our knowledge about IVDSCs remains largely limited.

Therefore, authors aim to perform a review to systematically discuss (1) the isolation, surface markers, classification, and biological characteristics of IVDSCs; (2) the aging- and degeneration-related changes of IVDSCs and the influences of IVD microenvironment on IVDSCs; and (3) the potential for IVDSCs to promote regeneration of degenerated IVDSCs. The authors believe that this review exclusively addresses the current understanding of IVDSCs and provides a novel approach for the IVD regeneration.

## 2. Identification of IVDSCs

In 2007, Risbud et al. identified a cluster of cells in human degenerated IVD that express the surface markers of SCs and could perform adipogenic, osteogenic, and chondrogenic differentiation [28]. Similarly, other studies also detected the cells presenting similar characteristics in all components of human IVD including the AF, NP, CEP, and putative SCN [6, 30, 32, 36]. These cells could be classified into MSCs according to the criteria established by the International Society for Cellular Therapy (ISCT) (for example, the plastic-adherent growth; the expression of CD105, CD73, and CD90 and the lack of expression of CD45, CD34, CD14 or CD11b, CD79alpha or CD19, and HLA-DR surface molecules; and multilineage differentiation ability *in vitro*) [30, 32, 37, 38]. From these reports, we can conclude that the endogenous SCs, also called IVDSCs, definitely exist within IVD. It has been isolated, not only from the human being but also from many other species including murine, rhesus macaque, porcine, and rabbit [6, 31, 39–42].

## 3. Classification of IVDSCs

**Figure 1:**
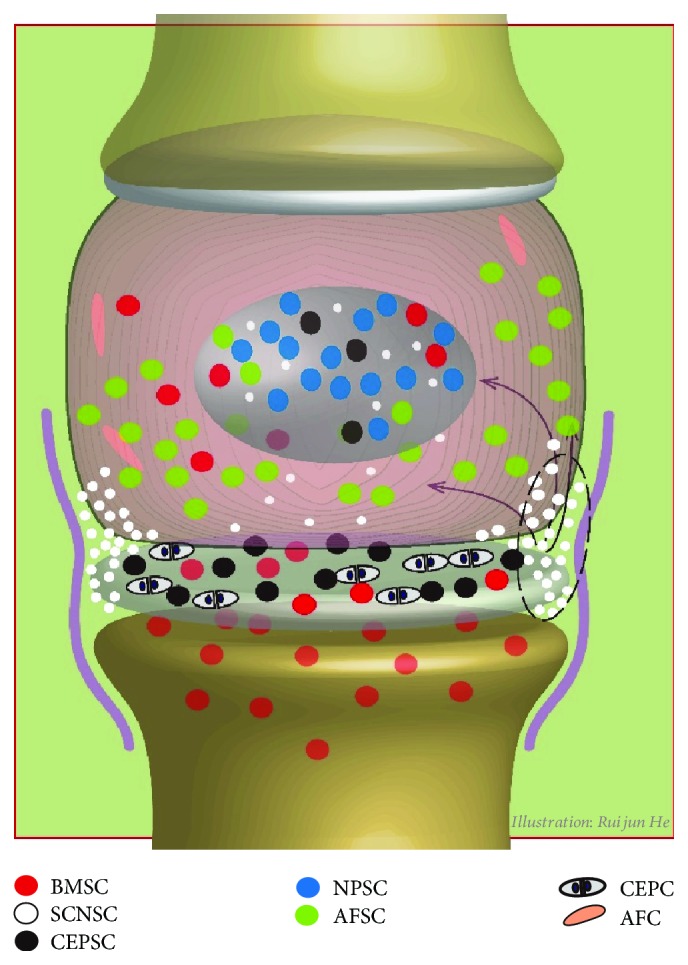
Schematic overview of the location of different kinds of IVDSCs. The stem cells located in IVD and the adjacent vertebras are indicated with dots. Elliptical broken line indicates the area of stem cell niche. The arrows indicate the possible migration pathways of SCNSCs. BMSC: bone marrow-derived stem cells; SCNSC: stem cell niche-derived stem cells; CESC: cartilage end plate-derived stem cells; NPSC: nucleus pulposus-derived stem cells; AFSC: annulus fibrosus-derived stem cells; CEPC: cartilage end plate cells. AFC: annulus fibrosus cells.

The normal IVD is composed of three distinct components: the central gelatinous NP, the outer AF, and the upper and lower CEP [43]. Based on the different anatomical regions of IVD, the IVDSCs are usually divided into three subsets, which are referred to as NP-derived stem cells (NPSCs), AF-derived stem cells (AFSCs), and CEP-derived stem cells (CESCs), respectively [28, 30, 36, 38]. Recently, some researchers propose the existence of SCN, a dynamic microenvironment consisting of the ECM and neighboring cells with the ability to regulate local SCs, within IVD [20, 27, 44]. Through a series of *in vivo* labeling procedures, the SCN is recognized as the perichondrium region adjacent to the EP and outer zone of the AF (Figure 1) [6, 20, 27]. Moreover, cells extracted from the SCN also meet the criteria defining MSCs [20]. Therefore, SCN-derived stem cells (SCNSCs) might be another classification of the IVDSCs [37].

However, classifying the IVDSCs into absolutely different four groups might not be completely reasonable when taking the sources of IVDSCs into account. Many researches have demonstrated that the SCs in SCN could migrate into the AF, NP, and CEP along certain routes [27, 44]. Our previous experiments also confirmed that the SCs in SCN could migrate into the inner part of IVD during the process of compression-induced IVD degeneration. Furthermore, results from Xiong et al. illustrated that the CESCs could migrate from CEP to NP tissue, and the migration could be inhibited by macrophage migration inhibitory factor [45]. In addition, the SCs might be infiltrated from growing vessels during disc degeneration, and adjacent bone marrow might also be the source in IVD [14, 46]. Despite the location of SCs in the different region, the SCNSCs, AFSCs, NPSCs, and CESCs possibly contain a proportion of cells having the same origin and biological characteristics. Hence, the SCs distributed in different anatomical regions of IVD are both independent and relevant. In Figure 1, we displayed the hypothetical distribution of IVDSCs according to different anatomic regions.

## 4. Isolation of IVDSCs

To date, many techniques have been developed to isolate stem/progenitor cells from different parts of the IVD. Generally speaking, most extraction methods are designed based on the distinct characteristics of SCs such as rapid proliferation rate, colony formation capacity, and unique surface markers [31, 38, 47, 48].

The SCs are characterized by their self-renew and rapid-proliferation capacity, which make them superior to other cells located in IVD in plastic-adherent and proliferation speed. Based on the above theory, differential adhesion method was developed to successfully isolate cells meeting the criteria of MSCs [47, 49, 50]. The main step of this method is to discard culture medium together with suspension cells and fragments when cell suspensions are seeded about 24 h later, and the remaining adherent cells are regarded as SCs without any further isolation [49]. Using this method, researchers have successfully isolated NPSCs from human and other species [11, 39, 47, 50, 51].

Colony formation is another important property of SCs. The cells with stem/progenitor cell characteristics survive at 50 cells/cm^2^, while other types of cells die due to loss of cell-cell contacts or undergo dissolution because of the low proliferation velocity [52]. Thus, colony formation assay might be another way to separate IVDSCs. Using this method, also named as limiting dilution method, Liu et al. extracted AFSCs from rabbit AF tissue, and they found an initial seeding density of 200 cells/cm^2^ to be optimal for the formation of colonies [33]. Similarly, rat NPSCs were also successfully isolated using this method [48]. For this method, the cell seeding density is the most critical because inappropriate cell density would cause the difficulty of colony formation or the failure of the SC extraction.

Some special culture medium is also utilized to isolate the IVDSCs. Agarose suspension culture is a chondrocyte selective culture system, in which chondrocytes are the only cell type to survive apart from tumor cells [48]. Employing the agarose culture system, the NPSC, AFSC, and CESC were all successfully isolated [32, 36, 38, 45, 53]. Methylcellulose semisolid medium, a culture system established to identify tissue-specific stem/progenitor cells from various organs, is also applied to extract the IVDSCs, and the NPSCs had been extracted through this method [31, 54]. In addition, some researchers including our group successfully extracted IVDSCs by using standard MSC expansion medium [55, 56].

Except for the above methods, the explant culture technique and fluorescence-activated cell sorter (FACS) cell sorting have also been developed to isolate IVDSCs [23, 31]. And some researchers even isolated IVDSCs directly by cell culture without any special treatment [30, 43, 57]. Besides that, some important points must be taken into consideration during the isolation of IVDSCs. Firstly, the blood cells must be thoroughly removed to avoid the contamination of SCs. Then, when isolating IVDSCs from human samples, the age and degeneration grade of patients must be taken into account, which might influence the quantity and quality of the IVDSCs [31]. So, the technique such as FACS cell sorting should be carried out to purify the IVDSCs meticulously.

## 5. Surface Marker of IVDSCs

The currently identified surface markers for IVDSCs are shown in Table 1. However, Sakai et al. demonstrated that some surface markers exist both in NP cells and NPSCs [31]. Thus, we must realize that the cells expressing these markers are not necessarily the IVDSCs. Therefore, it is quite exigent to explore the distinct surface markers of IVDSCs. From another aspect, the expression of surface markers is associated with functional status of the IVDSCs. For example, CD105 is related to cell migration, and the expression of Tie2 and GD2 indicates the differentiation of NPSCs [31, 32]. Additionally, the IVDSCs can express neural stem cell-associated surface markers. Some researchers have proven that AFSCs could perform neurogenesis differentiation and NPSCs could differentiate into Schwann-like cells [35, 54].

## 6. Biological Characteristics of IVDSCs

The biological characteristics of IVDSCs (given that the concept of SCNSCs is not received so far, we only discuss the NPSCs, AFSCs, and CESCs in this chapter) have been comprehensively explored. It has been displayed that all three kinds of IVDSCs share almost the same morphology and immunophenotype [30, 32, 34, 35, 38]. For proliferation capacity, Liang et al. demonstrated that NPSCs and AFSCs had stronger cell proliferation capacity than that of CESCs [30]. At the same time, results from Wang et al. exhibited that there was no significant difference in proliferation ability among NPSCs, AFSCs, and CESCs [38]. Additionally, for multilineage differentiation ability, Liang et al. concluded that the expression of different lineage differentiation-related genes of AFSCs was stronger than that of NPSCs and CESCs [30]. Wang et al. comprehensively compared the pluripotency of IVDSCs. They found that the osteogenic and chondrogenic capacities to be superior in CESCs followed by AFSCs and NPSCs; similarly, the adipogenic capacity to be superior in NPSCs followed by CESCs and AFSCs. However, when cultured in alginate bead, the CESCs consistently showed superior chondrogenic potential when comparing with rest of the cell types [38]. It seems that IVDSCs isolated from different anatomical regions have different biological characteristics. This phenomenon might be ascribed to the special microenvironment of different IVD components [38]. Furthermore, for the discrepancy in biological properties of IVDSCs among the studies, we speculate that this might be associated with different isolation technique, passaging, and culture protocols [38].

The IVDSCs also contain some special features that are different from other SCs. The CESCs are reported to have better osteogenic and chondrogenic ability as compared to BMSCs, while the NPSCs that were isolated from degenerated NP tissue showed much lower adipogenic differentiation ability [29, 32]. Wu et al. demonstrated that compared to umbilical cord mesenchymal stem cells (UCMSCs), NPSCs isolated from degenerated IVD displayed impaired proliferation capability and differentiation potential [23]. Furthermore, when cultured in a disc mimicking microenvironment, the NPSCs are more resistant to hypoxic and acidic pH microenvironment as compared to ASCs, making NPSCs preferable cell sources for IVD regeneration [11, 51].

## 7. The Aging- and Degeneration-Related Changes of IVDSCs

It is a proven fact that the disc cells undergo a series of biologic changes as they become old and degenerated. These changes include alternation of cell type in NP, decrease in number of viable cells, and increase in cell senescence [9]. Similarly, aging and degeneration also alter the quantity and quality of IVDSCs. With the progression of aging and degeneration, the number of cells expressing stem/progenitor cell markers in IVD tissues decreases markedly, indicating the exhaustion of IVDSCs [31, 41]. In Zhao et al.'s report, the aged NPSCs demonstrated deteriorative capacities of proliferation, colony forming, and multilineage differentiation but had more senescent features [40]. For degeneration-related changes, previous studies have shown that the NPSCs derived from degenerated NP have impaired ability in colony formation, chemotactic migration, proliferation, and have less expression of stem cell markers and stemness genes [42, 43]. In addition, these NPSCs exhibit notably inferior chondrogenic differentiation ability [42]. Owing to these aging- and degeneration-related changes, the failure of endogenous repair of IVD is inevitable. Thus, preventing or even reverting the changes incurred by aging or degeneration will be of great necessity in recovering or promoting the endogenous repair of IVD.

The DNA damage, telomere shortening, oxidative stress, and disturbance of the intracellular homeostasis contribute to the initiation of IVDD by causing cell senescence and programmed cell death [62–65]. With aging and degeneration, the degradation of misfolded proteins and clearance of toxic cellular waste products are constrained, making it difficult to maintain the homeostasis and resulting in a presenescence state [66, 67]. When the presenescence SCs are in quiescence, their intrinsic homeostasis can still be maintained [68]. However, once the presenescence SCs is activated to exert the endogenous repair function, it is difficult to maintain normal physiological activities and then die [69]. Therefore, repair capability of these subhealthy SCs can be restored by restoration of the cellular homeostasis, where autophagy may play a significant role. Sousa-Victor et al. reversed senescence and restored the regenerative properties of old muscle satellite cells in an injury model by promoting autophagy [69]. Sousa-Victor et al. reported that rapamycin, an agonist of autophagy, had antisenescence effects on AFSCs but inhibited the differentiation of AFSCs under multilineage induction, thus maintaining the stemness [69]. However, appropriate differentiation of SCs is also vital to IVD regeneration. So, precise regulation of the autophagy is required in order to keep the SCs in the right direction towards tissue repair.

## 8. The Influences of IVD Microenvironment on IVDSCs

The SCs are enclosed in a tissue-specific microenvironment that significantly influences their biological and metabolic vitality. The special microenvironment of the IVD is characterized by low oxygen tension, excessive stress or strain, hypertonicity, low pH, and poor nutrient supply, which present challenges to the survival and the function of implanted or endogenous SCs [70].

One overriding characteristic of disc cells is that they are resided under conditions of hypoxia due to the lack of blood supply. Under hypoxia, the NPSCs exhibit better cell proliferation ability than ASCs, and its chondrogenic capacity is enhanced when compared to normoxic environment [51]. For CESCs, hypoxic precondition might weaken the differentiation in osteogenic induction, indicating the antimineralization effect of hypoxia [60]. Thus, physiological hypoxia may be beneficial to exert normal physiological functions of IVDSCs. However, during the process of degeneration, blood vessels might invade into IVD through the fissures, which might increase the oxygen levels and disrupt the physiological hypoxic microenvironment of the IVDSCs [71, 72]. Therefore, restoring the hypoxic microenvironment may favor IVDSC-based endogenous repair of IVD.

In addition to hypoxia, excessive mechanical loading is another crucial microenvironmental factor. The disc-specific biomechanical features have been shown to exert a wide range of impacts on the biological functions of IVDSCs. *In vitro* studies have demonstrated that cyclic tensile stress would induce apoptosis of CESCs via the BNIP3/Bcl-2 pathway, whereas static compression stress would induce mitochondrial apoptosis in NPSCs [56, 59]. Besides the induction of cell death, stress stimuli are also essential for the normal function of SCs [73–75]. For example, it has been proven that the proportion of ECM components synthesized by AFSCs changes with fluid shear stress [76]. These results indicate the double-edged sword effects of mechanical loading. Therefore, further studies should focus on exploring various methods to protect IVDSCs from excessive mechanical loading-induced cell death and dysfunction. In the meanwhile, the biomimetic matrix would also be produced by optimizing mechanical stimulation for i*n vitro* cultured IVDSCs.

Moreover, hypertonicity, low pH, and poor nutrient supply are also important microenvironmental factors and are detrimental for implanted or endogenous SCs. The osmotic pressure of healthy NP, AF, and CEP (450~550 mOsm/L) is distinctly higher than the normal blood (280~320 mOsm/L) [77]. Nevertheless, the SC-related researches taking high osmolarities into consideration are still scarce. Tao et al. pioneered the investigation regarding the influence of osmotic pressure on IVDSCs [49]. They found that high osmolarity could decrease the viability, proliferation, and expression levels of SOX-9, aggrecan, and collagen II in NPSCs [49]. Regarding the influences of low pH, Han et al. compared ASCs with NPSCs which were cultured in an acidic environment [11]. They found NPSCs to be less inhibited in proliferation and cell viability [11]. Liu et al. further illuminated that in NPSCs, the acid-sensing ion channel (ASIC) plays an important role in acid-induced apoptosis and the downregulation in stem cell-related genes and ECM synthesis [55]. Furthermore, it was also demonstrated that the nutrition deficiency could cause mitochondrial translocation of BNIP3 in CESCs, leading to caspase-dependent apoptosis [78].

## 9. The Influences of ECM on IVDSCs

With the degeneration of IVD, the cell clustering and cell death make it more difficult to maintain the balance between anabolism and catabolism of ECM and further aggravate the tissue dysfunction [79, 80]. When investigating the IVDSCs *in vitro*, importance of ECM in the original tissue is often ignored. However, the cell-matrix interactions are essential in modulating not only the morphology and phenotype but also the function of SCs, and that has been successfully demonstrated in NP tissue [81, 82]. Perlecan, the common component of many SCNs, is found to be produced by progenitor cells located in this region [83–85]. In addition, it plays a positive role in chondrogenic differentiation of the mesenchymal progenitor cells in IVD [86, 87]. The changes in mechanical strength of the ECM in degenerated discs may be transmitted to the cell membrane and activate the IVDSCs via perlecan or other components of SCN [88, 89]. Moreover, the Piezo1 ion channel exists in human NP cells and underlies mechanical force-induced apoptosis via mediating the mitochondrial dysfunction and endoplasmic reticulum stress [90]. Similar mechanosensitive ion channels that function between ECM and cells are continued to be discovered. Nevertheless, whether there are other mechanisms in the modulation of stem cells' fate, and how the IVDSCs react with the alternation in mechanics, requires further studies.

Tissue engineering relies on suitable seed cells and scaffold materials. The biomechanical properties of scaffold materials affect the function of IVDSCs and determine the efficiency of IVD regeneration [91–94]. The natural molecules that make up the ECM of the IVD are a good choice of scaffold material. The laminin that exists in normal NP tissue, but is absent in degenerated NP tissue, attracts the attention of researchers [95–97]. Nerurkar et al. have presented a novel strategy using anisotropic nanofibrous laminates seeded with MSCs to replicate the form and function of the AF [98]. In addition, *in vitro* influences of laminins on the proliferation and chondrogenesis of SCs have also been demonstrated [99, 100]. Moreover, the SCs also produce laminins which in turn enhance the regeneration ability [101, 102]. Another important component of ECM in IVD is collagen II, which promote the differentiation of SCs, especially the chondrogenic differentiation in a concentration-dependent manner [103]. When cultured in a high concentration of collagen II, the ASCs express a high level of collagen II, aggrecan, SOX9, and low levels of collagen I, which is associated with cross-talk mechanisms between MAPK/ERK and Smad3 pathways [103, 104]. The small leucine-rich proteoglycans (SLRPs) are reported to be the key molecules in modulating the physiological and pathological process of SCs by binding to collagens, growth factors, and other matrix components in the niche of tendon [105] and articular cartilage [106], as well as IVD [107]. The SLRPs might act as a unique niche component regulating the activities of IVDSCs through hypoxia-inducible factor (HIF), thus allowing the survival of these cells under low oxygen tension [39]. In conclusion, the components of the ECM in IVD play a significant role in activation, self-renew, and differentiation of IVDSCs. However, the positive effects of these natural molecules on promoting IVD regeneration rely on optimistic space and time, which remains to be further elucidated [82, 108, 109].

## 10. The Potential of IVDSCs for IVD Regeneration

Endogenous neural stem cells react to stroke and spinal cord injury by generating a significant number of new neural cells [110]. In the brain, neural stem/progenitor cells might play a supportive role in the cortex to promote neuronal survival and glial cell expansion after traumatic brain injury [111]. Even more encouraging, using a surgical method preserving the endogenous lens epithelial stem/progenitor cells, Lin et al. successfully achieved the regeneration of functional lens in rabbits and macaques, as well as in human infants with cataracts [112]. Thus, motivating the IVDSCs to promote endogenous repair of IVD seems to be a prospective method for IVD regeneration. It has been proven that SCNSCs migrate toward and into IVD tissue following the intercellular space direction of the lamellae [44]. Huang et al. also proposed that stimulating endogenous SCs with simvastatin might retard the progression of IVDD [113]. Chen et al. further proved that transplantation of NPSCs has superior regenerative efficacy than transplantation of NP cells for treating IVDD in rabbit models [47]. However, current researches about the regenerative potential of IVDSCs are still scarce. Additionally, some endogenous factors might inhibit the migration of IVDSCs. For example, Xiong et al. have demonstrated that macrophage migration inhibitory factor secreted by NP cells could inhibit the migration of CESCs [45]. Therefore, further studies are desperately needed to explore the approaches of promoting the endogenous repair of IVD.

## 11. Conclusion

The IVD itself has endogenous stem/progenitor cells, which satisfy the criteria defining the MSCs. Compared to other SCs, the IVDSCs might be an excellent cell source for IVD regeneration due to the following advantages: (1) the IVDSCs are generally extracted from surgical specimens derived from patients with disc herniation. Thus, IVDSCs are more accessible and could avoid the damage caused by isolating other kinds of SCs, such as BMSCs. (2) IVDSCs are superior to other SCs in tolerating the harsh microenvironment of IVD.

However, there are some limitations in understanding of IVDSCs. First, current researches about IVDSCs are scarce. Therefore, we only have limited knowledge about the biological characteristics of IVDSCs. Then, surface markers of IVDSCs are still controversial and more specific markers are needed to identify IVDSCs *in vivo* and *in vitro*. Furthermore, how to isolate purer IVDSCs simply and economically is still waiting for exploration. Finally, how to protect IVDSCs from aging, degeneration, and harsh microenvironment is not fully elucidated. In conclusion, the IVDSCs might play a pivotal role in the regeneration of IVD, but more studies are necessary.

## Figures and Tables

**Table 1:** Surface markers of IVDSCs.

Species	Cell type	Positive markers	Negative markers	References
Human	NPSC	CD73, CD90, and CD105	CD34, CD45	[47]
Human	NPSC	CD73, CD90, and CD105	CD34, CD45, and HLA-DR	[43, 55]
Rat	NPSC	CD44, CD90, and CD105	CD34, CD45	[50]
Human	NPSC	CD29, CD44, and CD105	CD14, CD34, CD45, and HLA-DR	[34]
Human	NPSC	Tie2,GD2, Flt1, and CD271	CD24	[31]
Mini pig	NPSC	CD29, CD90, and CD44	—	[42]
Rat	NPSC	CD73, CD90, and CD105	CD34, CD45	[40]
Human	NPSC	CD29, CD44, CD73, CD90, and CD105	CD29, CD44, CD73, CD90, and CD105	[23]
Human	NPSC	CD90, CD73, CD105, CD106, and CD166	CD14, CD19, CD24, CD34, CD45, and HLA-DR,	[29]
Human	NPSC	CD24, CD73, CD90, and CD105	CD29, CD45	[58]
Rhesus macaque	NPSC	CD44, CD90, CD146, CD166, and HLA-DR	CD90, CD271	[39]
Human	AFSC, NPSC	CD49a, CD63, CD73, CD90, CD105, CD166, p75 NTR, and CD133/1	CD34	[28]
Rhesus macaque	AFSC	CD44, CD90, CD146, CD166, and HLA-DR	CD29, CD106, and CD271	[39]
Human	AFSC	CD29, CD49e, CD51, CD73, CD90, CD105, CD166, CD184, nestin, and neuron-specific enolase	CD31, CD34, CD45, CD106, CD117, and CD133	[35]
Human	AFSC, NPSC, and CESC	CD73, CD90, and CD105	CD19, CD34, CD45, and HLA-DR	[38]
Human	CESC	CD73, CD90, and CD105	CD14, CD19, CD34, CD45, and HLA-DR	[45, 59, 60]
Human	CESC	CD73, CD90, CD105, and Stro-1	CD14, CD19, CD34, CD45, and HLA-DR	[36]
Human	CESC	CD44, CD73, CD90, CD105, CD133, CD166, and Stro-1	CD14. CD19, CD34, CD45, and HLA-DR	[32]
Rat	SCNSC	CD29, CD90, and CD44	CD19, CD34, CD45, and CD11b	[20]
Human	IVDSC	CD90, CD105, and Stro-1	—	[61]
